# Common methods of measuring ‘informed choice’ in screening participation: Challenges and future directions

**DOI:** 10.1016/j.pmedr.2016.10.017

**Published:** 2016-10-28

**Authors:** Alex Ghanouni, Cristina Renzi, Susanne F Meisel, Jo Waller

**Affiliations:** Department of Epidemiology and Public Health, University College London, 1-19 Torrington Place, London, United Kingdom

**Keywords:** UK, United Kingdom, NHS, National Health Service, GMC, General Medical Council, IPDAS, International Patient Decision Aid Standards, Decision making, Research methodology, Mass screening

## Abstract

There is general agreement among public health practitioners, academics, and policymakers that people offered health screening tests should be able to make informed choices about whether to accept. Robust measures are necessary in order to gauge the extent to which informed choice is achieved in practice and whether efforts to improve it have succeeded. This review aims to add to the literature on how to improve methods of measuring informed choice. We discuss and critique commonly-used approaches and outline possible alternative methods that might address the issues identified. We explore the challenges of defining what information should be provided about screening and hence understood by service users, appraise the use of ‘thresholds’ to define e.g. positive attitudes towards screening, and describe problems inherent in conceptualising ‘informed choice’ as a single dichotomous outcome that either does or does not occur. Suggestions for future research include providing greater detail on why particular aspects of screening information were considered important, analysing knowledge and attitude measures at an ordinal or continuous level (avoiding problematic decisions about dichotomising data in order to set thresholds), and reconceptualising informed choice as a multifactorial set of outcomes, rather than a unitary one.

## Highlights

• There is ample scope to improve methods in studies on informed choice in screening.
• Researchers could be explicit regarding how their values shape their methods.
• There may be preferable alternatives to dichotomies of e.g. ‘adequate’ knowledge.
• It may be overly reductive to define ‘informed choice’ as a single entity.

## 1 Introduction

There is broad consensus in the United Kingdom (UK) that when people are invited to participate in health screening, they should make an ‘informed choice’ (Department of Health, 2011, National Screening Committee, 2013, General Medical Council, 2008). Individuals differ in how they appraise the balance of potential harms and benefits of screening, and hence whether they consider it worthwhile. This has led to a perceived ethical duty to encourage people to decide for themselves. For example, the National Health Service (NHS) in England informs people that “*deciding whether or not to have a screening test is a personal choice and one which only you can make*” (NHS Choices, 2015). To varying degrees this perspective is shared internationally (Andermann et al., 2008). The longstanding paternalistic view that screening communications should prioritise high levels of uptake has thus been superseded by a view that uptake can only be maximised within the constraints of informed choice. However, despite this consensus, there is a notable lack of agreement, consistency, and clarity about how informed choice should be defined and measured in practice (House of Commons Science and Technology Committee, 2015, Fox, 2006). The inevitable consequence is that efforts to improve it have made little progress (Biesecker et al., 2013).

Conceptual and methodological challenges are not necessarily apparent as authors usually have limited space to define informed choice and its underlying concepts. Descriptions are typically brief, e.g. “*an informed decision is one where all the available information about the health alternatives is weighed up and used to inform the final decision; the resulting choice should be consistent with the individual's values*” (Bekker et al., 1993). Similarly, “*an effective decision is one that is based on relevant knowledge, consistent with the decision-maker's values and behaviourally implemented*” (O'Connor and O'Brien-Pallas, 1989). Arguably, this gives the impression that informed choice is an uncomplicated interaction between i) a decision maker's knowledge about the proposed intervention; ii) their evaluation of the intervention with respect to their core values; and iii) their decision on whether or not to be screened (Marteau et al., 2001).

There are several ways in which informed choice is operationalised (see Biesecker et al., 2013 for examples) but this ‘trinity’ is often an instrumental aspect: researchers aim to measure screening invitees' knowledge using multiple-choice items or true/false statements (e.g. “*screening is for women without symptoms;*” Hersch et al., 2015). Values are measured in terms of participants' attitudes towards screening (e.g. “*some people find the test a bit unpleasant but it is simple to do and is designed to be done in the privacy of your own home. How does this make you feel about screening?*: *Against screening* |* Unsure* |* For screening*;” Smith et al., 2010). Screening behaviour can sometimes be determined by clinical records and screening intentions are often used as a proxy when it is not possible to measure behaviour directly (although this has well-recognised limitations; Sheeran, 2002). Thresholds are selected for each of these dimensions to categorise participants: knowledge may be labelled ‘good’, ‘adequate’, or ‘satisfactory’ vs. ‘poor’, ‘inadequate’, or ‘unsatisfactory’ and attitudes may be ‘positive’ vs. ‘negative’. Similarly, screening participation may be described in terms of having had or not had (or intending vs. not intending to have) the test. Participants are considered to have made an informed choice if they are categorised as having ‘adequate’ knowledge and behave in a way that is consistent with their values (e.g. if they have positive attitudes and undergo screening). Conversely, they are categorised as having made an uninformed choice if they are rated as having ‘inadequate’ knowledge or behave in a way that is inconsistent with their values. It should be acknowledged that informed choice overlaps with several related concepts (discussed below) such as broader umbrella terms like ‘informed decision-making’ (Sheridan et al., 2004, Briss et al., 2004) and more specific concepts within this (e.g. decisional conflict (O'Connor, 1995) and shared decision-making; Briss et al., 2004). However, the literature on informed choice contains numerous examples of the previously described approach to operationalise informed choice (e.g. Biesecker et al., 2013, Marteau et al., 2001, Hersch et al., 2015, Smith et al., 2010).

This narrative review draws on key studies in order to critique this method and suggest possible alternatives. Research on informed choice in screening is becoming more commonplace internationally and receiving greater attention from policymakers, particularly in the UK (House of Commons Science and Technology Committee, 2015). It is therefore crucial that methodologies are appropriate. We focus on three major issues. First, we discuss the challenge of defining what information is important for people when they are offered screening. Second, we consider the limitations inherent in setting thresholds for ‘adequate’ knowledge and ‘positive’ attitudes or intentions. Finally, we comment on the standard tripartite operationalisation of informed choice, described above. To our knowledge, we are the first to draw explicit, specific attention to these issues collectively and explore them in detail. We hope that this will contribute to discussions on how to ascertain whether informed choice is being achieved in screening. Our discussion is oriented around this context but our comments may also be applicable to other scenarios. For example, the same conceptualisation of informed choice is sometimes applied in areas such as shared decision-making and informed consent (e.g. Berger-Höger et al., 2015). Although these will not be the primary focus of this review and we will not address this literature directly, there are also no ‘gold-standard’ methods of measurement related to these concepts (Right Care Shared Decision Making Programme, 2012, Gillies et al., 2015).

## 2 Review

### 2.1 Defining important screening information and knowledge

The information that invitees might consider about screening participation includes exceptionally complex and multifaceted risks, benefits, and practical issues, comprising both conceptual and numerical components that are unfamiliar to most people. Statistics such as positive predictive value and the differences between survival vs. mortality are so challenging that an appreciable proportion of medical professionals misunderstand them (Wegwarth and Gigerenzer, 2013, Whiting et al., 2015). The potentially relevant information is even more complex in a screening setting that does not aim to identify a single disease but a range of diseases or risk factors, each with a unique set of risks and benefits resulting from detection (e.g. genomic screening; Elias and Annas, 1994). Consequently, an early step in the design of any study on informed choice is to make a judgement on which elements constitute pertinent information to those offered screening, in order to decide which aspects of their knowledge to assess. Generally, researchers aim to complete this step by attempting to follow recommendations of published guidelines or the stated preferences for information among (potential) screening service users. However, both approaches have important limitations.

There are several sets of applicable guidelines; one of the most prominent in the UK is published by the General Medical Council (GMC), which states that screening invitees should be provided with the information that they “*want or need about*… *the potential benefits, risks and burdens, and the likelihood of success, for each option*” (General Medical Council, 2008). These recommendations are broad in order to apply to a wide range of medical decision-making contexts, meaning that they lack specific suggestions on what invitees should be informed of when deciding whether to have a screening test. However, it is notable that the recommendation that communicators “*should not make assumptions about the information a patient might want or need*” is not compatible with many organised screening programmes, in which the entire eligible population typically makes screening decisions after being provided with identical information materials, without speaking to health professionals. These guidelines have similarities to another set published by the International Patient Decision Aid Standards (IPDAS) Collaboration for improving ‘decision quality’ (Elwyn et al., 2006). These also include standards relating to what information should be conveyed to people being asked to make a healthcare decision, although in some respects these are more detailed (e.g. “*use event rates specifying the population and time period*”). Previous studies of informed choice have been guided by both sets of recommendations (e.g. Marteau et al., 2001, Smith et al., 2010, Smith et al., 2009, Michie et al., 2002, Kellar et al., 2008).

We consider it debateable whether there is a clear reason to favour any particular set of guidelines. Ostensibly, they offer the benefit of reflecting a consensus among experts “*based on a rigorous assessment of the evidence base*” (National Institute for Health and Care Excellence, 2012). However, guidelines are not only based on the available scientific evidence, but also the cultural and personal values of the experts and other individuals appraising that evidence (Kelly et al., 2015, Bekker, 2010). Indeed, this is an acknowledged component of the systematic Delphi process used to design the IPDAS checklist (Elwyn et al., 2006). This should be considered when attempting to use guidelines as a gold standard to decide what constitutes important information. To illustrate this, although the IPDAS checklist explicitly recommends that a decision aid should include information on “*detection*/*treatment that would never have caused problems if one was not screened*”, the perceived importance of this is influenced by the personal values of a given researcher or health service provider. Individuals will differ in terms of whether they believe this represents information about screening that is essential, useful for participants to be aware of but not crucial, unnecessary, or undesirable since it would cause participants undue burden and fear (Jepson et al., 2005, Parker et al., 2015a, Parker et al., 2015b).

The most common alternative to guidelines is to define important information based on the views of (potential) screening service users. However, in a survey of older UK adults, the large majority stated a personal preference for “*all the information currently available*” on the risks and benefits (Waller et al., 2012). The complexity of medical literature makes this effectively impossible (Elias and Annas, 1994); researchers have to balance providing ‘full’ information with avoiding overwhelming invitees. Furthermore, it is problematic to define what ‘full’ information would consist of (see Manson and O'Neill, 2007 for a more in-depth exploration of these points in the context of informed consent). Members of the public also vary in their stated preferences for information: previous research has found that some advocate either limiting information on the risks of screening or omitting it entirely, on the basis that its inclusion would decrease participation (Woodrow et al., 2008). Conversely, researchers may believe there is an ethical duty to offer this information (Independent UK Panel on Breast Cancer Screening, 2012). These examples highlight how it is often necessary for researchers to interject their own judgements on whether to design information that aligns with the preferences of service users or to override them based on practical or ethical considerations.

The preceding comments have focused on information provision. However, this alone is insufficient for ‘*informed*’ choice, which also requires that information is absorbed and internalised in a meaningful (and, ideally, demonstrable) way. There are also challenges in this respect: there is little apparent agreement regarding the appropriate level of ‘granularity’ to knowledge about screening. Invitees may be expected to understand a concept (“*screening saves lives from breast cancer*”) without necessarily being aware of the underlying statistics and their implications (e.g. “*screening saves about 1 life from breast cancer for every 200 women who are screened*;” National Breast Screening Programme, 2013). Thus, on the basis of expert advice, a previous study elected not to provide participants with statistics relating to breast cancer screening (van Agt et al., 2012), whereas other authors have recommended their inclusion (e.g. Spiegelhalter et al., 2011, Baum, 2006, Barratt et al., 2005). There has also been some explicit recognition that knowledge can exist at different ‘levels’. For example, Reyna (2008) distinguishes between knowledge based on ‘verbatim’ and ‘gist-level’ information and others have assessed knowledge in both conceptual and numerical terms (e.g. Hersch et al., 2015, Smith et al., 2010, Mathieu et al., 2007). This was also an important issue in the design of the revised information leaflet for the Breast Screening Programme in England, in which there were evident tensions between the views of academics and potential service users. The former group generally recommended detailed explanations to illustrate applicable scientific caveats whereas the latter group found it distracting (Forbes et al., 2014).

A further challenge is determining what constitutes ‘accurate’ knowledge, which is particularly problematic when there is little consensus among academics or clinicians. In the case of breast screening, the estimated magnitudes of benefits (in terms of reductions in breast cancer mortality) and harms (in terms of overdiagnosis) are highly variable (Paci et al., 2014, Gøtzsche and Nielsen, 2013), with the extensive epidemiological literature increasing rather than reducing uncertainty (Independent UK Panel on Breast Cancer Screening, 2012). In such “*contested terrain*”, researchers measuring informed choice must decide which responses will be characterised as correct or incorrect. They also have to consider the extent to which they inform participants about any lack of consensus. These decisions will almost always be open to challenge (Sasieni et al., 2015).

The lack of clearly superior guidelines, methods of accounting for public stated preferences, objectively appropriate levels of granularity to knowledge, and unambiguous evidence defining ‘accurate’ knowledge is reflected in review findings. There is a striking lack of consistency among studies that aim to improve informed choice in terms of what information was provided to participants and how much detail was given (Biesecker et al., 2013). There is similar inconsistency regarding which aspects of knowledge are measured (Mullen et al., 2006). In summary, the complexity of screening gives rise to i) an inherently value-based and ideological component to determining whether knowledge of a given piece of screening information might be considered important for informed choice; ii) numerous possible ‘levels’ at which screening information might be retained as knowledge; and iii) varying degrees of empirical uncertainty regarding what would constitute ‘accurate’ knowledge. However, we believe that there are alternative approaches that would mitigate these issues considerably.

### 2.2 Thresholds for dichotomising measures

The two most common methodological approaches for setting thresholds for ‘adequate’ (or ‘good’) knowledge are to either use a standard based on observed data (e.g. a median split; Marteau et al., 2001, Kellar et al., 2008) or predetermined value such as a scale midpoint (e.g. Smith et al., 2010, Michie et al., 2002, Mathieu et al., 2007). Less commonly used approaches include multifactorial coding schemes (e.g. correctly answering 50% of items, including one numerical item on three knowledge subscales; Hersch et al., 2015).

First, it is difficult to justify defining ‘adequate’ or ‘good’ knowledge using post-hoc thresholds based on observed data, since descriptions like ‘adequate’ imply an absolute standard, whereas the location of (e.g.) a median is relative to the observed data (Marteau et al., 2001). Second, to our knowledge, there are no robust criteria by which ‘adequate’ knowledge could reasonably be inferred from a specific number of items answered correctly. As with the issues around defining ‘important’ information, decisions around where and how to set thresholds are inherently subjective.

These limitations are not specific to informed choice (Altman and Royston, 2006) but they are more prominent since they are often a formal part of how it is operationalised. Variability in thresholds and lack of validation has previously been documented in this context (Biesecker et al., 2013, Ames et al., 2015), and the problem is illustrated clearly by van Agt et al. (2012): a sensitivity analysis in which the threshold for ‘sufficient’ knowledge was varied between 8 and 13 out of 13 knowledge items answered correctly found that the proportion of participants categorised as having made an informed choice ranged from 19% to 88%.

Related to the challenges outlined previously, these issues are exacerbated since knowledge items are not perceived as either strictly ‘important’ or ‘not important’ but rather important to varying degrees. Although it is analytically convenient to give equal weighting to each item, this is unlikely to be a reflection of the perceptions of any given reader of a study (or participant). For example, those who consider overdiagnosis to be the main harm of screening are unlikely to assign equal importance to items that measure understanding of this concept compared with items measuring understanding of false positive results. This is not taken into account when the same score is applied to all knowledge items. These same considerations are likely to apply to measures of attitudes and intentions in that thresholds are often defined for e.g. ‘positive’ attitudes using the same problematic approaches, which also overlook differences in the relative importance of items.

On one level, these issues appear to be well known: in 2001, Marteau and colleagues noted that “*the terms good and poor imply an absolute standard against which knowledge is judged. It is intended that such a standard is developed in future*” (Marteau et al., 2001). However, in 2015, Hersch et al. state that “*no consensus exists on what level of knowledge constitutes being informed*” (Hersch et al., 2015). Similarly, Ames et al. conclude that “*particular attention ought to be directed towards addressing how ‘good’ knowledge and informed choice are defined and measured from the outset of a screening programme*” (Ames et al., 2015). Unfortunately, it appears that there has been little progress in the past 15 years. As with the difficulties in determining what knowledge should be considered relevant to people offered screening, we are not convinced that it is realistic to aim for either an empirically-derived standard of ‘adequate’ knowledge (or ‘positive’ attitudes and intentions), or a standard based on expert opinion that could not be legitimately disregarded by researchers with a different perspective. As above, we believe that there are alternative approaches that would lessen these issues considerably.

### 2.3 Standard operationalisations of informed choice

As Jepson et al. note, it is open to debate whether informed choice can be defined and measured meaningfully, although this is a necessary assumption of research into the concept (Jepson et al., 2005). We agree that this assumption is not self-evidently true and we believe there are some clear parallels between informed choice and cultural constructs that are rarely thought of as measurable. For example, two individuals could discuss a notion like ‘justice’ in much the same way as informed choice, given that there is a clear public interest in ensuring justice in criminal trials. Hence, they might construct a seemingly plausible and coherent definition as the basis of a measure (e.g. “*a just outcome is one where the jury weighs up all the necessary evidence and uses it to inform the final verdict; sentencing of a defendant found guilty should be consistent with society*'*s values*”; adapted from Bekker et al., 1993). The next step would be to operationalise it in order to categorise a given verdict as ‘just’ or ‘unjust’. The multiplicity of competing viewpoints of what constitutes important evidence would have to be addressed, as would the challenges of determining whether the jury's knowledge of the evidence (comparable to knowledge of screening information) was ‘adequate’ or ‘good’, and quantifying society's values in terms of favouring punishments that are ‘severe’ vs. ‘lenient’ for a given crime (analogous to positive vs. negative attitudes).

In this analogy, we believe that informed choice resembles justice very closely. Despite the desirability of a measure, both concepts are extremely abstract, value-laden, and open to multiple interpretations, so operationalisations in which they are treated as a single entity are difficult to support, especially those in which informed choice (or justice) is defined as a discrete event that either occurs or does not, detached from the sociocultural context. Hence, we do not take for granted that informed choice is an observable, empirical phenomenon rather than a more complex philosophical idea. Indeed, as far as we are aware, the extensive psychological research on the topic of justice itself has focused on measuring different levels of *perceived* justice across various dimensions (e.g. ‘procedural’ and ‘interpersonal’ justice), rather than attempting to define and measure it based on an objective standard (Colquitt et al., 2001).

### 2.4 Future directions

Per our own collective values, we believe that informed choice ought to be an integral aspect of screening policy in the UK and internationally but we also believe there should be a reappraisal of the underlying concepts and methods on which studies rely. In this section, we explore alternative approaches that we hope might overcome the previously discussed issues. We do not discuss relevant standards of methodological quality (e.g. confirming reliability and validity) as they apply independently of our suggested approaches.

First, in order to mitigate difficulties in defining what constitutes ‘important’ information, we recommend following the example of some authors (e.g. Hersch et al., 2015, Kellar et al., 2008, van Agt et al., 2012, de Haan et al., 2013), who report results of individual items that measure knowledge, rather than only aggregate measures of the overall number of items answered correctly. This offers greater transparency and allows a reader to interpret results based on their personal view of the importance of specific aspects of screening knowledge. It will often be useful to sum the number of correct responses to create an overall score. However, we suggest that this should generally be given a lower priority and only reported in addition to the results of individual items (and never instead of them). A similar rationale may apply to reporting of measures of attitudes.

Given that selecting aspects of information to provide and aspects of knowledge to assess requires researchers to draw from their own values, we also suggest they are as explicit as possible about why selected topics were considered important. For example, if researchers select items based on particular guidelines, it is worth clarifying the rationale for using that set and the process used to map specific recommendations on to knowledge items. Similarly, it would be informative to explain how items were selected based on input from (prospective) screening participants, if applicable. Researchers could also clarify whether any perceived practical or ethical considerations meant the final set of knowledge items did not fully reflect guidelines or services users' stated preferences, and highlight limitations of the selected approach (e.g. whether conceptual rather than numerical understanding of particular information was measured). Finally, research evidence used to determine ‘correct’ responses to knowledge items should be cited clearly (e.g. Hersch et al., 2013). These measures will allow a reader to gain a fuller understanding of the process underpinning the study design, even if they do not agree with it.

We have also argued that dichotomising measures of knowledge, attitudes, and intentions is difficult to justify on empirical or theoretical grounds. Subjective and arbitrary thresholds may be addressed by taking into account the natural properties of the data (for example, that the number of correctly answered knowledge items is continuous). Analyses that reflect this are likely to be more robust and would also address the additional problem that information is lost when data are dichotomised (Altman and Royston, 2006). It may also allow more nuanced and informative statistical approaches. For example, regression analyses could test whether higher knowledge scores are associated with more or less positive attitude scores, whether more positive attitude scores are positively associated with screening participation (Marteau et al., 2001), and possibly whether knowledge or attitude has a stronger association with participation. It would also allow hypothetical moderation effects to be tested: more positive attitude scores may be associated with a greater probability of screening participation but this association may be smaller among participants with more knowledge of harms. This could be extended with respect to our first suggestion of reporting individual items: moderation effects could be relevant where researchers believe that a single, specific aspect of knowledge has particular ethical or empirical significance (e.g. overdiagnosis in breast cancer screening; Hersch et al., 2015).

Furthermore, although we have focused on a particularly common method of measuring informed choice, there are other factors that may be considered relevant and are not encapsulated by knowledge, values, and behaviour (Biesecker et al., 2013). In their 2006 review, Mullen et al. (2006) use a broader conceptualisation of ‘informed decision-making’ rather than informed choice, which they defined in terms of people's understanding of the nature of a disease and the healthcare service available (i.e. risks, benefits, alternatives, and uncertainties), their evaluation of the risks and benefits with respect to their values, and engagement in decision-making at a level they find personally desirable (Sheridan et al., 2004, Briss et al., 2004). This mapped on to a more diverse range of measures in the context of cancer screening, including decisional self-efficacy, role preference, and decisional conflict. There may also be additional topics of interest within each of these. To take the latter example, the Decisional Conflict Scale is a widely used, validated measure that comprises three subscales with the aim of assessing the “*state of uncertainty about the course of action to take*” (O'Connor, 1995): decision uncertainty, perceived effective decision-making, and factors contributing to uncertainty. In addition, each subscale contains items that relate to further issues that may be considered important. For example, one item measures the extent to which an offer of screening is free from coercion (“*I feel pressure from others in making this decision*”). These might be used in addition to or instead of standard components of informed choice. Mullen et al. (2006) highlight a shortcoming of studies prior to 2006 that a conceptual framework for informed decision-making was often lacking; they advocate the use of frameworks and theory as one method of mitigating heterogeneity in variables measured. In some respects, the operationalisation that we have addressed is consistent with this recommendation and so represents an improvement over pre-existing measures (often of knowledge alone). However, we believe that it would be more useful for screening policy if it were broadened at least as far as incorporating outcomes relating to informed decision-making.

We also suggest that research could be improved if the three components of informed choice were evaluated individually, rather than attempting to unify them into a single overall ‘construct’. A more wide-ranging and less reductive conception of informed choice might also lead researchers to consider novel hypotheses. For example, since screening invitees are generally asked to consider information in the context of complicated social and cultural dynamics between themselves and a healthcare provider (Manson and O'Neill, 2007), studies might assess the association between the level of trust in those offering screening and uptake of the test (Entwistle et al., 2008). Studies might also assess how long knowledge is retained, since this relates to how long it remains valid to assess recall after a choice is made, and whether there are any ‘side-effects’ of encouraging an informed choice (e.g. greater fear of overdiagnosis or false positives). Table 1 contains a summary of our suggestions.

The following example illustrates some practical ways in which these methods might benefit policymakers and healthcare providers: a healthcare provider might commission a randomised controlled trial to compare a new information leaflet that aims to better inform invitees about the risk of overdiagnosis in breast screening (cf Forbes et al., 2014) with the current leaflet (i.e. “usual care”). Measures might include knowledge of various aspects of screening (e.g. overdiagnosis and potential health benefits), attitudes towards screening, and uptake. Results that follow from our suggestions might indicate that relative to the control arm, the intervention demonstrates: i) superior knowledge of overdiagnosis and comparable knowledge of other aspects of screening; ii) more negative attitudes towards screening; iii) comparable uptake. This would reassure policymakers that the leaflet improves knowledge of overdiagnosis, without adversely affecting population health outcomes via reduced uptake (all else being equal). However, the more negative attitudes towards screening may represent a concern that should be addressed. This relatively rich evaluation of the effects of an intervention contrasts with the same hypothetical study in which the primary outcome is a dichotomous measure of informed choice. In this case, the greater proportion of participants with knowledge of overdiagnosis, specifically, might not be reflected in a greater proportion of people with a level of knowledge over an arbitrary threshold. Furthermore, the more negative attitudes towards screening and comparable levels of uptake might imply that fewer people are making an informed choice, and obscure the possibilities that attitudes are not strongly associated with screening behaviour (a hypothesis that could be tested in the first example). Some previous studies have used or advocated certain aspects of the first approach (e.g. Marteau et al., 2001, Hersch et al., 2015, Smith et al., 2010) and we believe this shows how a more detailed appraisal would more meaningfully inform policymakers and healthcare providers than a reductionist approach.

In closing, we acknowledge that following our suggestions would have some limitations. First, reporting more outcomes may raise potential statistical issues around testing multiple hypotheses but we would argue that these are well-recognised and relatively straightforward to address in comparison to the more problematic conceptual issues that we believe would likely be resolved. Second, undertaking more complicated analyses and interpreting a broader range of outcomes may be more challenging and time-consuming than other methods. We would counter that they are likely to be more useful to policymakers and healthcare providers in the long-term if they offer more validity. However, a potential threat to this usefulness is if there is significant divergence between researchers' conceptions of (e.g.) the information they believe to be important and the views of service users and policymakers. This may lead to study results being irrelevant or impossible to compare. However, we are not suggesting an entirely individualistic approach to defining important topics but one in which service users, healthcare providers, policymakers, academics, and other stakeholders continue to shape the views of one another, which is likely to minimise disagreement. We would also argue that the methods we have suggested are not creating issues themselves but rather adding transparency around issues that are inherent in the topic.

We have focused on quantitative methods of studying informed choice because we believe there are significant benefits to generating information that is readily interpretable and actionable. Although we hope that quantitative approaches can still be justified, one might extend our arguments to the conclusion that informed choice is ultimately too subjective to be amenable to this approach. This might imply that an even more abstract conceptualisation may be warranted. For example, policymakers may promote the value of informed choice as a cultural norm among practitioners, the effects of which might suit qualitative methods of assessment. We offer our suggestions as a contribution to future discussions for improving the measurement of informed choice.

## 3 Conclusion

We have discussed some important limitations of commonly used approaches to measuring informed choice, which suffer from a lack of agreed standards regarding which aspects of knowledge are important, subjective or inappropriately defined thresholds for dichotomising key components, and an overly reductive conceptualisation. We suggest some alternative approaches that may be more appropriate. Instead of attempting to define informed choice as either occurring or not occurring, we suggest reporting detailed results using individual items, explicit descriptions of how information and items were selected, avoiding the use of thresholds, greater use of regression methods to explore associations between components, and more wide-ranging relevant measures. We hope our suggestions will foster discussions on how informed choice research can be developed, as this will allow more valid auditing of the extent to which informed choice is achieved in practice, and lend greater weight to comparisons of interventions that aim to improve screening.

## Funding

The current article was supported by a programme grant from Cancer Research UK awarded to Prof Jane Wardle (C1418/A14134). Cancer Research UK was not involved in the writing of the manuscript; or in the decision to submit for publication.

## Contributors

AG conceived this review. All authors participated in drafting and critical revision of the manuscript, and approved the final version.

## Conflicts of interest

No conflicts of interest have been declared.

## Transparency document

Transparency document.

## Figures and Tables

**Table 1:** Suggested future directions for research.

• Report data on knowledge in terms of how participants respond to items on individual topics; place less emphasis on aggregated data (e.g. the number of items answered correctly) • Detail explicit reasons why information topics were selected e.g. why particular guidelines or types of service user input were used and not others, how selection was affected by practical constraints or researchers' values • Refer to the evidence underpinning how responses to knowledge items were classified as correct or incorrect • Analyse ordinal and continuous data without setting problematic thresholds for dichotomisation • Analyse data using more informative statistical methods (such as regression, allowing e.g. moderation effects to be tested) • Consider dimensions relevant to informed choice beyond knowledge, values, and behaviour e.g. aspects of decisional conflict • Report data on knowledge, values, and behaviour (and behavioural intentions) separately, without aggregating them into a single, overall variable labelled ‘informed choice’ • Consider hypotheses that would be unfeasible to test using existing conceptualisations of informed choice (e.g. whether knowledge of screening predicts uptake)
